# The Mosses of Crocker Range Park, Malaysian Borneo

**DOI:** 10.3897/phytokeys.88.14674

**Published:** 2017-10-11

**Authors:** Monica Suleiman, Dunstan Polus Masundang, Hiroyuki Akiyama

**Affiliations:** 1 Institute for Tropical Biology and Conservation, Universiti Malaysia Sabah, Jalan UMS, 88400 Kota Kinabalu, Sabah, Malaysia; 2 Museum of Nature and Human Activities & Phylogenetic Division, Institute of Natural and Environmental Science, Hyogo Prefectural University, Yayoigaoka-6, Sanda, Hyogo 669-1546, Japan

**Keywords:** Bryophytes, CRP, Crocker Range, East Malaysia, Sabah

## Abstract

This paper reports the mosses from Crocker Range Park (CRP) in Sabah, Malaysian Borneo. In total, 293 species, three subspecies and eight varieties belonging to 118 genera and 36 families are reported. This represents about 40% and 47% of the species and infra-specific taxa reported from Borneo and Sabah, respectively. Out of these, six species are new records for Borneo, namely *Barbella horridula*, *Chaetomitrium lancifolium*, *Distichophyllum leiopogon*, *Rhaphidostichum luzonense*, *Rosulabryum capillare* and *Taxiphyllum taxirameum* and 12 species and one variety are new to Sabah. With these additions, the current number of mosses in Sabah and Borneo are 651 and 766, respectively. The largest family of mosses is Calymperaceae with 35 species and one subspecies, followed by Sematophyllaceae with 32 species and two varieties and Pylaisiadelphaceae with 21 species and one variety. In conclusion, CRP has a very high species richness of mosses which is the second highest in Borneo, after Mount Kinabalu.

## Introduction

Crocker Range Park (CRP) is located in the west coast of Sabah, East Malaysia in Borneo (latitude 5°07' to 5°56'N and longitude 115°50' to 116°28'E). This park is about 110 km long and 15 km wide, covering an area of 139,919 ha, making it the largest terrestrial park and protected area in Sabah. This park was first designated as a Forest Reserve under the Forest Ordinance in 1969 but was subsequently converted to a State Park in 1984 for the conservation of natural resources and ecosystems, under the jurisdiction of Sabah Parks Trustees (Usui et al. 2006). In June 2014, Crocker Range was designated as a UNESCO Biosphere Reserve consisting of the whole area of CRP and the three forest reserves within the range.

CRP, in the past, had received less attention from bryologists when compared to Kinabalu Park. These two parks are both on the Crocker Range which is the longest range in Sabah, extending from Kudat (northern tip of Borneo) to Sipitang (southern part of Sabah). CRP has become more accessible after the establishment of seven substations within the park between the years 2003 and 2005 and the opening of a new road system from Ulu Kimanis (western part) to Keningau Town (eastern part), cutting through the central part of the park. Another factor which may have contributed to the lesser attention received by CRP is the fact that its highest peak is only 2,076 m a.s.l., just half of that of Mount Kinabalu (4,059 m a.s.l.). Nevertheless, 27% of the total area of CRP is more than 1,000 m a.s.l., with 16 peaks above this height (Usui et al. 2006).

To date, only two studies on mosses from this park have been published. Suleiman and Akiyama (2004) reported 126 species of mosses belonging to 74 genera and 27 families, collected during the CRP Scientific Expedition in 2002 at Ulu Kimanis and adjacent areas within the elevations of 500–1,400 m a.s.l. Recently, Suleiman and Jotan (2015) reported 38 species and three varieties of mosses belonging to 17 genera and 11 families collected during a diversity study of epiphytic mosses along the Minduk Sirung Trail, a new 12 km trail connecting Mount Alab and Mahua substations (north-eastern part). In their study, mosses were collected from only three sampling areas of 20 m × 20 m.

There are two other unpublished studies on mosses in CRP. The first one was by Kong (2006), who conducted a study on the diversity of mosses in Keningau Research Permanent Plot which is only 50 m × 50 m. She collected 40 species belonging to 26 genera and 14 families. The second one was by Chin (2008), who has studied the diversity of epiphytic mosses within 0–2 m of tree trunks, in the Mount Alab Permanent Research Plot (50 m × 50 m). She collected 20 species in 10 genera and seven families in this mossy forest (1,700–1,800 m a.s.l.). The present report attempts to produce a comprehensive checklist of mosses found in CRP based on collections from the year 2002 to 2008 and herbarium specimens deposited in the BORNEENSIS Herbarium of the Institute for Tropical Biology and Conservation, Universiti Malaysia Sabah (BORH) and Herbarium of Museum of Nature and Human Activities, Hyogo (HYO).

## Methods

**Figure 1.**
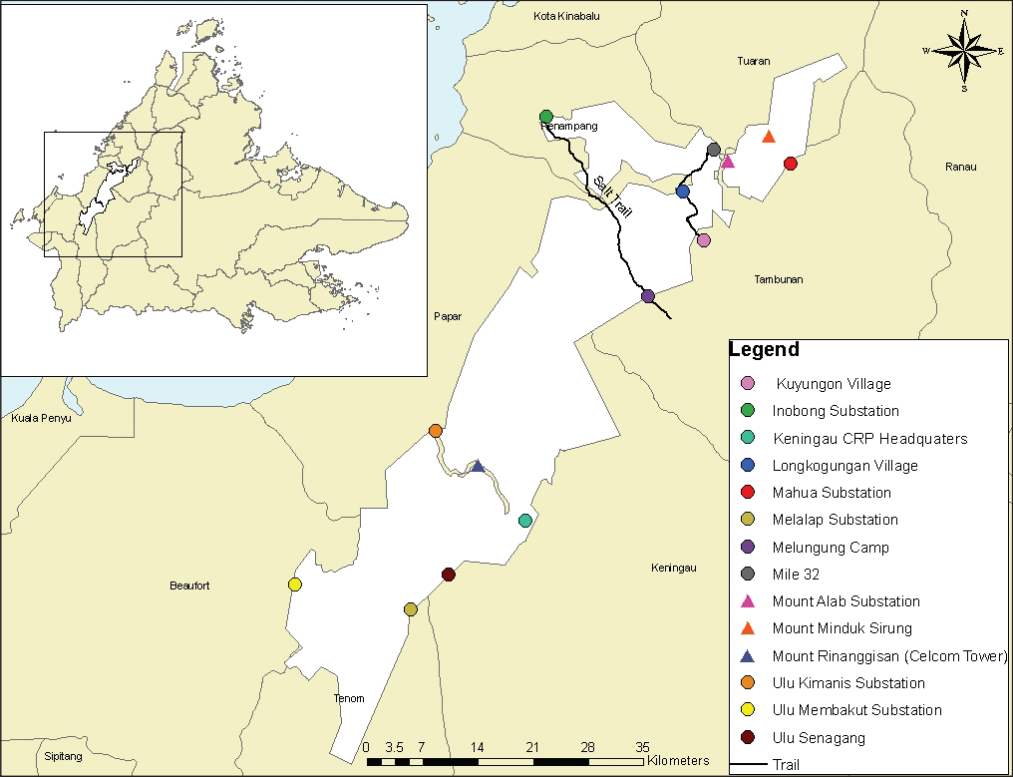
Map of Crocker Range Park showing the localities of collections from the year 2002 to 2008. Inset is map of Sabah, Malaysian Borneo.

All specimens of mosses from the following 12 localities within the park were examined and identified. Areas covered are Inobong Visitor and Research Station, Mount Alab, Mile 32-Longkogungan Village, Longkogungan-Kuyungon Village, Salt Trail, Mahua Substation, Mount Minduk Sirung, CRP Headquaters, Ulu Senagang Substation, Melalap Substation, Ulu Membakut Substation and Ulu Kimanis Substation (Figure 1). These localities range from lowland to upper montane forests, covering secondary to primary forests, from 50 m to 2,000 m a.s.l. Details of the collection localities are listed in Table 1. Identified specimens were deposited at BORH and a set of duplicates were sent to the Herbarium of Sabah Park (SNP). Some duplicates were also deposited at HYO, Herbarium of University of Malaya (KLU) and Herbarium of Royal Botanic Gardens Victoria (MEL).

**Table 1.** Locality and collection details of mosses from Crocker Range Park from the year 2002 to 2008. CMK - Chin Mui Ken; DPM - Dunstan Polus Masundang; HA-Cr - Hiroyuki Akiyama-Crocker; KWL - Kong Wai Ling; MS - Monica Suleiman.

Collection numbers	Locality
MS 877–1006	Ulu Kimanis, Mt. Rinangisan and surrounding areas, 5°28.15'N, 116°03.53'E, 27–30 Aug. 2002.
HA-Cr 1–467	Ulu Kimanis, CRP Headquaters and Mt. Alab pass, 27 Aug.–15 Sept. 2002.
MS 1182–1244	Mahua Waterfall, 5°49.60'N, 116°23.11'E, 8-23 July 2003.
MS 1245–1263	Salt Trail, Tikolod to Inobong, 5°39.62'N, 116°15.49'E to 5°51.51'N, 116°8.33'E, 23–28 Aug. 2003.
MS 1386–1391	Mahua Waterfall, Nature Trail and trail to Minduk Sirung, 5°49.60'N, 116°23.11'E, 12–13 Dec. 2003.
MS 1406–1407	Mt. Alab, above Gunung Emas Restaurant, 14 Dec. 2003.
MS 1430–1461	Tenom, Melalap, trail to Tarangtali Hill, along Mesisilad River and Kallang Waterfall, 27–29 Jan. 2004.
KWL 1–96	CRP Headquaters, Permanent Research Plot, 5°23.97'N, 116°06.16'E, 12 Oct. 2005.
MS 1488–1489	Mt. Alab, Permanent Research Plot, 5°49.31'N, 116°20.49'E, 5 July 2006.
CMK 1–163	Mt. Alab, Permanent Research Plot, 5°49.31'N, 116°20.49'E, 24–25 May 2007.
MS & DPM 2357–2434	Mt. Alab, around Mt. Alab Garden, 5°49.31'E, 116°20.49'E, 13 Dec. 2007.
DPM 2–112	Mt. Alab, vicinity of Mt. Alab Substation and Permanent Research Plot, 5°49.31'N, 116°20.49'E, 19–20 Jan. 2008.
MS & DPM 2533–2712	Mahua, Mt. Minduk Sirung, 5°49.60'N, 116°23.11'E, 1–3 Apr. 2008.
DPM 126–180	Ulu Senagang, trail to waterfall and along park boundary, 5°21.78'N, 116°1.72'E, 28–29 Aug. 2008.
DPM 181–258	Inobong Visitor and Research Station, trail to waterfall and Buayan Village, 5°51.51'N, 116°8.33'E, 1–2 Sept. 2008.
MS & DPM 3776–3877	Mt. Alab, vicinity of Mt. Alab Substation, 5°49.31'N, 116°20.49'E, 8–10 Sept. 2008
MS & DPM 3878–3936	CRP Headquaters, Permanent Research Plot & Crocker Trail, 5°23.97'N, 116°06.16'E, 11 Sept. 2008.
DPM 259–318	Ulu Membakut, along Membakut River and park boundary adjacent to Inantul Village, 5°20.97'N, 115°54.06'E, 18–20 Sept. 2008.
MS & DPM 3937–4057	Longkogungan and Kuyungon Village, 5°49.97'N, 116°19.33'E to 5°42.56'N, 116°19.33'E, 22–23 Sept. 2008.
MS & DPM 4058–4095	Bolotikon Village to Melungung Camp, 25 Sept. 2008.
MS 4123–4130	Ulu Kimanis, Mt. Rinangisan and Permanent Research Plot, 5°28.15'N, 116°03.53'E, 13–14 Nov. 2008.
MS 4131–4136	CRP Headquaters, Permanent Research Plot, 5°23.97'N, 116° 06.16'E, 19 Dec. 2008.

## Results and discussion

A total of 1,403 specimens of mosses from CRP were examined during this study. Amongst these, 293 species, three subspecies and eight varieties belonging to 118 genera and 36 families were identified (Table 2 and Appendix 1). The five dominant families of mosses in CRP are Calymperaceae with 35 species and one subspecies (11.8%), followed by Sematophyllaceae with 32 species and two varieties (11.2%), Pylaisiadelphaceae with 21 species and one variety (7.2%), Dicranaceae with 21 species (6.9%) and Daltoniaceae with 20 species (6.6 %). All of these families, except for Dicranaceae, are lowland families as ca. 70% of CRP land area is below 1,000m a.s.l.

**Table 2.** Mosses reported from Crocker Range Park (See Appendix 1 for species checklist).

No.	Families	Genera	Species, subspecies and varieties
1.	Bartramiaceae	1	3 spp.
2.	Brachytheciaceae	3	4 spp.
3.	Bryaceae	4	7 spp.
4.	Calymperaceae	7	35 spp. and 1 subsp.
5.	Cryphaeaceae	1	1 sp.
6.	Daltoniaceae	6	20 spp.
7.	Dicranaceae	8	21 spp.
8.	Diphysciaceae	1	3 spp.
9.	Ditrichaceae	1	1 sp.
10.	Entodontaceae	3	3 spp.
11.	Fissidentaceae	1	13 spp. and 1 var.
12.	Garovagliaceae	1	4 spp. and 1 var.
13.	Hookeriaceae	1	1 sp.
14.	Hypnaceae	6	12 spp.
15.	Hypnodendraceae	3	6 spp.
16.	Hypopterygiaceae	3	4 spp.
17.	Leskeaceae	2	2 spp.
18.	Leucobryaceae	6	16 spp. and 2 var.
19.	Leucomiaceae	1	1 sp.
20.	Meteoriaceae	7	11 spp.
21.	Mniaceae	1	3 spp.
22.	Myuriaceae	1	1 sp.
23.	Neckeraceae	7	14 spp.
24.	Orthotrichaceae	2	7 spp.
25.	Pilotrichaceae	4	5 spp.
26.	Polytrichaceae	2	8 spp. and 2 subsp.
27.	Pottiaceae	3	4 spp.
28.	Pterobryaceae	7	9 spp.
29.	Pylaisiadelphaceae	6	21 spp. and 1 var.
30.	Racopilaceae	1	3 spp. and 1 var.
31.	Regmatodontaceae	1	1 sp.
32.	Rhizogoniaceae	2	4 spp.
33.	Sematophyllaceae	10	32 spp. and 2 var.
34.	Sphagnaceae	1	3 spp.
35.	Symphyodontaceae	2	4 spp.
36.	Thuidiaceae	2	6 spp.
	Total	118	293 spp., 3 subsp. and 8 var.

The species richness of mosses in the study area is very high; 40% of the total of 766 species and infra-specific taxa reported from Borneo and 47% of the total of 651 species and infra-specific taxa reported from Sabah (Andi and Suleiman 2005, Suleiman et al. 2006, 2009, 2011a, 2011b, 2017, Suleiman and Akiyama 2007, Higuchi et al. 2008, Akiyama 2010, Ho et al. 2010, Ellis et al. 2010, 2015, 2016a, 2016b, Andi et al. 2015, Chua and Suleiman 2015, Mohamed et al. 2010, Suleiman and Rimi 2016).

Out of the 293 species, three subspecies and eight varieties of mosses in CRP, six are new to Borneo and 13 are new to Sabah (Table 3). Amongst the six species new to Borneo, four were found in the lowland areas between 70 m and 680 m a.s.l. Lowland areas in Borneo have not been given enough bryological attention, probably due to the misconception that the lowland rainforest has poor species richness of bryophytes. For instance, *Chaetomitrium lancifolium*, which was collected at 70 m a.s.l. in CRP, represents a second known record after its type collection from the Maluku Islands (see Appendix 1 for details).

**Table 3.** New records of mosses to Borneo and Sabah.

No.	Moss species and variety	New records	
		Borneo	Sabah
1.	*Acroporium macroturgidum*		+
2.	*Acroporium ramicola*		+
3.	*Barbella horridula*	+	+
4.	*Chaetomitrium lancifolium*	+	+
5.	*Clastobryum scalare*		+
6.	*Distichophyllum leiopogon*	+	+
7.	Leucobryum javense var. cyathifolium		+
8.	*Leucobryum juniperoideum*		+
9.	*Papillidiopsis malayana*		+
10.	*Rhaphidostichum luzonense*	+	+
11.	*Rosulabryum capillare*	+	+
12.	*Schoenobryum concavifolium*		+
13.	*Taxiphyllum taxirameum*	+	+
	Total	6	13

Several of the mosses found in CRP are of temperate entities and rarely reported in Borneo, namely *Claopodium prionophyllum*, *Elmeriobryum philippinense*, *Entodon plicatus*, *Erythrodontium squarrosum*, *Leucomium strumosum*, *Mesonodon flavescens*, *Oxyrrhynchium vagans*, *Pseudoleskeopsis zippelii*, *Regmatodon declinatus* and *Schoenobryum concavifolium*. Five of these species, namely *Claopodium prionophyllum*, *Entodon plicatus*, *Erythrodontium squarrosum, Mesonodon flavescens* and *Oxyrrhynchium vagans*, have only been collected once in Borneo (Dixon 1916, Iwatsuki and Noguchi 1975, Akiyama et al. 2001). *Elmeriobryum philippinense* was collected during the study and reported as new to Borneo by Ellis et al. (2016a). In addition, three species endemic to Borneo were also found in this park: *Benitotania elimbata*, *Ectropothecium ptychofolium* and *Acroporium ramicola* (Appendix 1).

Crocker Range Park ranks the second highest (cf. Table 4) in terms of number of mosses reported from mountainous areas in Borneo (Frahm et al. 1990, Suleiman and Edwards 2002, Suleiman and Akiyama 2004, Higuchi et al. 2008, Akiyama et al. 2001, Andi et al. 2015, Suleiman et al. 2011b). CRP recorded about 40% of the mosses reported from Borneo although the highest point in CRP is only 2,076 m a.s.l. This indicates that CRP has high species richness of mosses, second to that of Mount Kinabalu. Meanwhile, the number of mosses on Mount Trus Madi and Mount Lumaku were much lower, with 26% and 17%, respectively. Although Mount Trus Madi is much higher in terms of elevation, the number of mosses reported from the mountain was far lower than from CRP. Mount Lumaku, on the other hand, has a similar height to the highest peak of CRP but its species richness is only about half that of CRP. Two of the contributing factors are that CRP receives a high annual rainfall and it has a relatively larger area of pristine primary lowland forests than Mount Trus Madi and Mount Lumaku. Nonetheless, a diversity study should be carried out to determine the true diversity of these areas.

**Table 4.** Moss species and infra-specific taxa reported from mountainous areas in Borneo.

Geographical area	Elevation Range (m a.s.l.)	Number of moss species and infra-specific taxa	% of moss species and infra-specific taxa
Kinabalu Park	600-4,095	386	51
Crocker Range Park	50-2,076	304	40
Mount Trus Madi	600-2,642	194	26
Mount Lumaku	700-1,966	130	17

## Conclusion

CRP is a huge protected area and large parts of this park have not been surveyed during the present study. Thus, additional explorations in less accessible areas will definitely increase the number of mosses in this park and provide a better understanding of the distribution of species within the park. The large area of lowland forests in CRP is an asset to this protected area as it harbours important species of mosses and other plants. Large areas of lowland forest in other parts of Borneo have been cleared for agriculture and development, adding to the importance of conservation of this UNESCO Biosphere Reserve. This study identifies CRP as one of the hotspots of moss diversity in Borneo.

## Appendix 1

Checklist of mosses from Crocker Range Park.

The families, genera and species were arranged in alphabetical order. Species reported for the first time for Sabah and Borneo are marked with ‘*’ and ‘**’, respectively. CMK - Chin Mui Ken; DPM - Dunstan Polus Masundang; HA-Cr - Hiroyuki Akiyama-Crocker; KWL - Kong Wai Ling; MS - Monica Suleiman.

**Bartramiaceae**

*Philonotis bartramioides* (Griff.) D.G. Griffin & W.R. Buck

On boulders by river banks and road sides, 500–1580 m, DPM 128; MS & DPM 3939.

*Philonotis hastata* (Duby) Wijk & Margad.

On boulders, 385 m, MS 1447, 1448, 1458.

*Philonotis secunda* (Dozy & Molk.) Bosch & Sande Lac.

On soil by road sides and along trails in partially shaded and open areas, 680–1800 m, HA-Cr 140; MS 927; MS & DPM 3814, 3873

**Brachytheciaceae**

*Oxyrrhynchium vagans* (A. Jaeger) Ignatov & Huttunen

On a rock by a river, 1020 m, MS 1199.

*Rhynchostegiella vriesei* (Dozy & Molk.) Broth.

On a tree trunk, 940–1120 m, HA-Cr 263.

*Rhynchostegium celebicum* (Sande Lac.) A. Jaeger

On rotten logs and rocks, 400–1030 m, DPM 184; MS 1210.

*Rhynchostegium javanicum* (Bél.) Besch.

On a wet rock beside waterfall, 980–1100 m, HA-Cr 292.

**Bryaceae**

*Brachymenium nepalense* Hook.

On fallen logs, and tree and shrub trunks, 1150–1400 m, HA-Cr 191, 359; MS 946; MS & DPM 4033.

*Bryum apiculatum* Schwägr.

On soil and boulders, 650–1800 m, MS & DPM 3875, 3991; HA-Cr 210.

*Bryum clavatum* (Schimp.) Müll. Hal.

On concrete in open area by road-side, 900 m, MS & DPM 3912.

*Bryum coronatum* Schwägr.

On crevice by a road-side in open area, 1370 m, MS 925.

*Rhodobryum aubertii* (Schwägr.) Thér.

On rock by a river, 1030 m, MS 1215, 1219.

*Rosulabryum rubens* (Mitt.) J.R. Spence

On soil in an open area, 50 m, DPM 295.

***Rosulabryum capillare* (Hedw.) J.R. Spence

On rotten log by a stream, 1600 m, MS & DPM 2695.

Plants yellowish-red, forming lax tufts, 1.5 cm tall, matted with rhizoids at base. Leaves large, flaccid, spatulate, 2.0–2.7 mm × 0.5–0.7 mm; apex broad, rounded with an abruptly long piliform apiculus, arista 0.4–0.6 mm long, coloured; costa reddish, very strong at base, attenuate towards apex; margins revolute, plane 1/3 above, denticulate in apical region, strongly bordered throughout by 1–4 rows of elongated cells, strongly thick-walled, reddish, Mid lamina cells rhomboidal, 49–54 μm × 17–25 μm, thin-walled, rectangular towards leaf base. Sporophyte not seen.

This species is almost cosmopolitan in distribution but is not common in Malesia where it has been recorded previously only from New Guinea, the Philippines and Malaya. It is easily identified by the spatulate leaves with broadly and rounded apex and an abruptly long piliform apiculus as illustrated by Eddy (1996).

**Calymperaceae**

*Arthrocormus schimperi* (Dozy & Molk.) Dozy & Molk.

On humus, rotten logs, tree trunks and tree bases, 80–1145 m, DPM 200, 278, 311; KWL 51; MS 1445; MS & DPM 4064.

*Calymperes afzelii* Sw.

On boulder by river, open area, 550 m, MS & DPM 3996.

*Calymperes boulayi* Besch.

On tree trunk in open area, 100 m, DPM 317.

*Calymperes fasciculatum* Dozy & Molk.

On a tree trunk, 1280 m, MS & DPM 3956.

Calymperes lonchophyllum subsp. beccarii (Hampe) M. Menzel

On rocks, roots and tree bases by streams, 70–100 m, DPM 259, 292, 308.

*Calymperes porrectum* Mitt.

On boulder and tree bases by streams, 100–680 m, DPM 135, 268; HA-Cr 154.

*Calymperes robinsonii* B.C. Tan & W.D. Reese

On boulders and stone-wall by rivers, 410 m, MS 1457.

*Calymperes serratum* A. Braun ex Müll. Hal.

On shrub trunks, 680–1600 m, HA-Cr 141; MS & DPM 2675.

*Calymperes strictifolium* (Mitt.) G. Roth

On tree trunks and tree bases, 680 m, HA-Cr 139.

*Calymperes taitense* (Sull.) Mitt.

On tree root, 500 m, DPM 139.

*Exostratum blumii* (Nees ex Hampe) L.T. Ellis

On boulders, roots, tree trunks and tree bases, 400–1400 m, DPM 194, 198, 202; HA-Cr 61, 257, 351, 395.; KWL 126a, 22b, 23, 25a, 26, 34, 43, 52a, 53a, 56, 60, 62, 84, 90, 93, 100b, 101, 114.

*Exostratum sullivantii* (Dozy & Molk.) L.T. Ellis

On a tree trunk, 1310 m, MS 981.

*Leucophanes angustifolium* Renauld & Cardot

On stone-walls, boulders, roots, tree trunks and bases, 100–1025 m, DPM 131, 155, 160, 162, 271; MS 1209; MS & DPM 3926, 3982.

*Leucophanes candidum* (Schwägr.) Lindb.

On rotten logs and tree trunks by river banks, 100 m, DPM 272, 313, 318.

*Leucophanes octoblepharioides* Brid.

On boulders, rotten logs, tree trunk, roots and tree stump, 80–1230 m, DPM 163, 165, 179, 180, 207, 267, 310; KWL 22a, 23, 25c, 93; 126b; HA-Cr 138, 389; MS 1435; MS & DPM 2533.

Mitthyridium fasciculatum (Hook. & Grev.) H. Rob. subsp. fasciculatum

On rotten logs, treelet trunks and river bank, 100–1220 m, DPM 274; HA-Cr 181; MS 997.

Mitthyridium fasciculatum subsp. cordotii (M. Fleisch.) B.C. Tan & L.T. Ellis

On rotten and fallen logs, and tree trunks, 550–1145 m, DPM 147; HA-Cr 427; MS & DPM 3883.

*Mitthyridium repens* (Harv.) H. Rob.

On decaying logs, tree trunks and tree bases, 400–800 m, DPM 205, 221; HA-Cr 121.

*Mitthyridium subluteum* (Müll. Hal.) H.K. Nowak

On climber, 1220 m, MS 996.

*Mitthyridium undulatum* (Dozy & Molk.) H. Rob.

On tree trunk beside streams, 950–1050 m, KWL 20a; MS 1212; MS & DPM 3932.

*Octoblepharum albidum* Hedw.

Growing on rotten logs, tree trunks and tree bases, 50–900 m, DPM 204, 222, 249, 281, 304; HA-Cr 317; MS 1431, 1434; MS & DPM 4013, 4014.

*Syrrhopodon albo-vaginatus* Schwägr.

On tree trunks and bases, and rotten logs, 50–1145 m, DPM 181, 156, 305; HA-Cr 422; KWL 95.

*Syrrhopodon aristifolius* Mitt.

On tree trunks and rotten logs, 650–1145 m, DPM 244, 257; KWL 91.

*Syrrhopodon ciliatus* (Hook.) Schwägr.

On rotten logs by river, 80–100 m, DPM 273, 309, 315, 316.

*Syrrhopodon confertus* Sande Lac.

On tree ferns, palm trees, tree trunks, tree bases and roots 610–1145 m, HA-Cr 423; KWL 2, 25b, 122; MS & DPM 3878.

*Syrrhopodon croceus* Mitt.

On rotten logs, 50–900 m, DPM 299; HA-Cr 202.

*Syrrhopodon gardneri* (Hook.) Schwägr.

On rotten logs, decaying logs, tree trunks and tree bases, 1100–1800 m, DPM 79; HA-Cr 326; MS 885; MS & DPM 3816, 3881.

*Syrrhopodon involutus* Schwägr.

On rotten logs, 705 m, MS 1432.

*Syrrhopodon japonicus* (Besch.) Broth.

On soil, climbers, tree trunks, bases and buttress, tree stumps and rotten logs, 1100–1800 m, HA-Cr 83, 91, 198, 321; MS 899, 924; MS & DPM 2547, 2615, 3830, 3950, 4030.

*Syrrhopodon laevis* (Dixon) Mohamed & W.D. Reese

Growing on rotten logs and tree trunks, 1700–1800 m, CMK 58, 52; DPM 25; MS & DPM 2616, 3791, 3817.

*Syrrhopodon loreus* (Sande Lac.) W.D. Reese

On roots, buttress, tree trunks and bases, 100–750 m, DPM 201, 266, 270; MS 4129; MS & DPM 4071.

*Syrrhopodon muelleri* (Dozy & Molk.) Sande Lac.

On tree trunks and bases, 1100–1300 m, HA-Cr 96; KWL 104; MS & DPM 2538, 3908.

*Syrrhopodon prolifer* Schwägr.

On soil, tree trunk and tree bases, 600–1800 m, DPM 229; CMK 84, 158; HA-Cr 363; MS & DPM 2706.

*Syrrhopodon spiculosus* Hook. & Grev.

On rotten logs, 600–1200 m, DPM 225; HA-Cr 87.

*Syrrhopodon tjibodensis* M. Fleisch.

On decaying logs, climbers and tree trunks, 1350–1800 m, MS 908, 919, 948; MS & DPM 3781, 4046.

*Syrrhopodon tristichus* Nees ex Schwägr.

On humus, tree stumps, rotten logs, shrub trunks and branches, tree branches and roots, 1370–1810 m, DPM 5, 67; HA-Cr 15, 199; MS 886, 923; MS & DPM 2541, 2624, 3855, 3942, 4043.

**Cryphaeaceae**

**Schoenobryum concavifolium* (Griff.) Gangulee

On concrete in an open area, 800 m, MS & DPM 4054.

This species has been reported as new to Borneo based on a collection from Kalimantan (Akiyama, 2012).

**Daltoniaceae**

*Achrophyllum javense* (Dixon ex J. Froehl.) Z. Iwats., B.C. Tan & Touw

On a wet boulder at streambed, 1600 m, MS & DPM 2693.

*Benitotania elimbata* H. Akiyama, T. Yamag. & Suleiman

On tree trunks, 1800 m, MS & DPM 3825.

*Calyptrochaeta parviretis* (M. Fleisch.) Z. Iwats., B.C. Tan & Touw

On tree trunks, rotten logs, boulders and shrub branches, 680–1425 m, HA-Cr 150, 399, 408, 410; MS 961; MS & DPM 3898.

Calyptrochaeta cf. ramosa (M. Fleisch.) B.C. Tan & H. Rob.

On the base of tree trunk, 1300 m, HA-Cr 63, det. B.C. Ho.

It has all the characteristics of the species but the leaf border has 3–4 rows of elongated cells instead of 2–3 rows.

*Calyptrochaeta remotifolia* (Müll. Hal.) Z. Iwats., B.C. Tan & Touw

On fallen logs and boulders, 770–1800 m, HA-Cr 280; MS & DPM 3864, 4084.

*Daltonia armata* E.B. Bartram

On rotten logs, bamboo stump and tree trunks, 750–1350 m, HA-Cr 111; MS & DPM 3897, 3907, 4031, 4095.

*Daltonia contorta* Müll. Hal.

On shrub trunks and branches, 1150–1400 m, HA-Cr 14, 192, 358.

*Distichophyllum acuminatum* Bosch & Sande Lac

On shrubs, 1240–1360 m, HA-Cr 30a, 277, 346.

*Distichophyllum catinifolium* J. Froehl.

On tree trunk and bases beside a stream, 1160 m, HA-Cr 396.

*Distichophyllum cirratum* Renauld & Cardot

On rocks, rotten logs and soil, 1100–1700 m, HA-Cr 29, 31, 32, 354, 355, 356, 375; MS & DPM 3854, 3892.

*Distichophyllum cuspidatum* (Dozy & Molk.) Dozy & Molk.

On tree trunk and branches, shrub trunks and decaying logs, 1150–1800 m, CMK 163; DPM 58, 96a, 98, 108; HA-Cr 30b, 33, 74, 196, 336; MS 976; MS & DPM 2597, 2625, 3826.

***Distichophyllum leiopogon* Dixon

Growing on soil in partially shaded area, 1700 m, MS & DPM 3853, det. B.C. Ho.

Leaves crisped when dry, spathulate, 3.0 mm × 1.2–1.3 mm, apex rounded to obtuse, with a small mucro, costa reaching 3/4 of leaf length, margin entire, border with 1–3 of cell. Lamina cells rectangular to hexagonal, 40–50 μm × 15–32 μm, thin walled. Calyptra smooth, fringed at base. Seta 7 mm, papillose throughout.

This species was previously recorded in the Philippines and New Guinea (Ho et al. 2010). Its cells near the leaf margin are only slightly smaller than the paracostal regions, distinguishing it from other species with spathulate or obovate leaf shapes.

*Distichophyllum malayense* Damanhuri & Mohamed

On fallen decaying tree trunks and rotten logs, 750–1800 m, HA-Cr 381, 357; MS & DPM 3858, 4040, 4073, 4075, 4077, 4080.

*Distichophyllum mittenii* Bosch & Sande Lac.

On rotten logs and tree roots, 750–1880 m, HA-Cr 7, 285, 353; MS 986; MS & DPM 2652, 2681, 2696, 3844, 4037, 4072, 4076, 4087.

*Distichophyllum nigricaule* Mitt. ex Bosch & Sande Lac.

On moist to wet rocks by streams 560 m, HA-Cr 304, 313.

*Distichophyllum osterwaldii* M. Fleisch.

On moist to wet rocks and boulders, and rotten logs, 750–1800 m, HA-Cr 100, 286, 377, 385; MS & DPM 3862, 4079.

*Distichophyllum subcuspidatum* Nog. & Z. Iwats.

On trunk of a shrub, 1512 m, MS 921.

*Distichophyllum spathulatum* (Dozy & Molk.) Dozy & Molk.

On a rotten log, 1127 m, MS 1392.

Distichophyllum cf. tortile Dozy & Molk. ex Bosch & Sande Lac.

On a rotten log, 750 m, MS & DPM 4081.

This specimen has all the characteristics of the species except for its leaf border which consists only of 1–2 rows of cells. Commonly, the species has 2–3 rows of cells.

*Ephemeropsis tjibodensis* K.I. Goebel

On palm, tree and shrub leaves by rivers, 750–1700 m, HA-Cr 20; MS & DPM 2666, 3843, 3887, 3976, 4074.

**Dicranaceae**

*Braunfelsia dicranoides* (Dozy & Molk.) Broth

On tree trunk, tree base and humus, 1100–1200 m alt, HA-Cr 84, 182, 222.

*Braunfelsia edentula* (Mitt.) Wijk & Margad.

On humus and shrub trunks, 1730–1800 m, MS & DPM 2606, 3803.

*Braunfelsia plicata* (Sande Lac.) Broth.

On fallen log, 1200–1730 m, MS & DPM 3836, 4025.

*Campylopus ericoides* (Griff.) A. Jaeger

On boulders and soil, 500–1000 m, DPM 212, 213, 216; MS & DPM 3993, 4053.

Campylopus exasperatus (Nees & Blume) Brid. var. exasperatus

On soil, 1800 m, DPM 110.

Campylopus fragilis subsp. zollingerianus (Müll. Hal.) J.-P. Frahm

On soil in an open area, 1150 m, HA-Cr 178.

*Campylopus laxitextus* Sande Lac.

On rotten branches and logs, and humus on tree bases, 1080–1800 m, MS & DPM 3815, 3900, 4049; HA-Cr 51, 187, 360.

*Campylopus serratus* Sande Lac.

On soil and rotten logs, 500–700 m, DPM 217; MS & DPM 4062.

*Campylopus umbellatus* (Schwägr. & Gaudich. ex Arn.) Paris

On soil, crevice, gravel, concrete and humus, 900–1800 m, HA-Cr 22, 56, 208; MS 926, 952; MS & DPM 3801, 3813, 3938.

*Cryptodicranum armittii* (Müll. Hal.) E.B. Bartram

On tree trunk, 1700–1800 m, MS & DPM 2603, 3828.

*Dicranella setifera* (Mitt.) A. Jaeger

On wet soil in open areas, 680–1800 m, HA-Cr 113, 426; MS & DPM 3874.

*Dicranoloma assimile* (Hampe) Paris

On tree buttress, trunks and roots, rotten logs and soil, 1160–1760 m, MS 889, 1257; MS & DPM 2554, 2566; 2635, 2636, 2638, 3948, 3951, 4028.

*Dicranoloma billardierei* (Brid.) Paris

On humus and rotten logs, 1720–1800 m, MS & DPM 2610, 2640, 3821, 3792.

*Dicranoloma blumii* (Nees) Paris

On trunks of shrubs and trees, 1100–1800 m, CMK 42, 151; HA-Cr 92; MS & DPM 2565, 2569, 3787.

*Dicranoloma braunii* (Müll. Hal.) Paris

On shrub and tree trunks, tree bases, roots and rotten stumps, 1080–1800 m, CMK 33, 123; KWL 59; MS & DPM 2536, 2560, 2605, 2621, 2622, 2671, 3905, 3937.

*Dicranoloma brevisetum* (Dozy & Molk.) Paris

On tree and shrub trunks, rotten logs and climbers, 1150–1870 m, CMK 33; CMK 131; DPM 3, 6, 81; HA-Cr 215, 320, 369; MS & DPM 2564, 2580, 2585, 2593, 2598, 2649, 2687, 3789, 3798, 3856, 4039.

*Dicranoloma reflexum* (Müll. Hal.) Renauld

On fallen log, 1100 m, MS 1387.

*Holomitrium cylindraceum* (P. Beauv.) Wijk & Margad.

On fallen log, 1090 m, MS 1388.

*Leucoloma molle* (Müll. Hal.) Mitt.

On boulders, tree and shrub trunks, 680–1600 m, HA-Cr 48, 155, 240; KWL 110; MS 1239, 1240, 2673, 3888, 4023.

*Leptotrichella brasiliensis* (Duby) Ochyra.

On rock by road side, 1340 m, MS 956.

*Leptotrichella miqueliana* (Mont.) Lindb. ex Broth.

On soil of trail banks, 680–950 m, HA-Cr 425; MS & DPM 3929.

**Diphysciaceae**

*Diphyscium foliosum* (Hedw.) D. Mohr

On wet rock, 1400 m, HA-Cr 349.

*Diphyscium longifolium* Griff.

On rocks, 1200–1340 m, MS 974, 985, 1393.

*Diphyscium mucronifolium* Mitt.

On rocks and wet boulders, 560–1700 m, HA-Cr 42, 160, 161, 171, 308, 347; MS 938, 939; MS & DPM 3848, 3891.

**Ditrichaceae**

*Garckea phascoides* Müll. Hal.

On road banks in sunny and open areas, 680–800 m, HA-Cr 116.

**Entodontaceae**

*Entodon plicatus* Müll. Hal.

On soils, boulders, rocks, tree branches and rotten logs, 600–1130 m, MS 1194, 1221, 1243; MS & DPM 3994, 4006.

*Erythrodontium squarrosum* (Hampe) Paris

On a shrub trunk by a road side, 900 m, MS & DPM 3911.

*Mesonodon flavescens* (Hook.) W.R. Buck

On concrete, boulders and tree trunks in open areas, 600–800 m, MS & DPM 4003, 4005, 4055, 4057.

**Fissidentaceae**

*Fissidens ceylonensis* Dozy & Molk.

On rocks, wet boulders and soil, 900–1700 m, HA-Cr 411; MS 975a; MS & DPM 3847, 3849, 3916.

*Fissidens crassinervis* Sande Lac.

On rocks, 1370 m, MS 884.

Fissidens crenulatus var. elmeri (Broth.) Z. Iwats. & Tad. Suzuki

On termite mount and rocks, 1080–1145 m, KWL 111, 124; MS & DPM 3902.

Fissidens crispulus Brid. var. crispulus

Growing on rocks, roots, boulders, stone-walls, soils and rotten logs, 100–1120 m, DPM 117, 149, 182, 264, 282, 269; MS 1441; HA-Cr 125, 256, HA-Cr 429; MS 1450, 1460, 3978, 4059, 4069.

Fissidens crispulus var. robinsonii (Broth.) Z. Iwats. & Z.H. Li

On rock, soil and stone-wall by streams and road side, 100–680 m, DPM 133, 286, 287; HA-Cr 126.

*Fissidens geppii* M. Fleisch.

On wet boulders and rocks by waterfall, and on soil in disturbed area, 900–1120 m, HA-Cr 247, 250; MS & DPM 3921, 3925.

*Fissidens hollianus* Dozy & Molk.

On tree branches, rotten logs, boulders and tree bases, 400–1600 m, DPM 132, 203; MS & DPM 2698, 2700, 3917.

*Fissidens hyalinus* Wilson & Hook.

Growing on wet rocky cliff and rocks by waterfall, 900–1100 m, HA-Cr 293; MS & DPM 3924.

*Fissidens javanicus* Dozy & Molk.

On soil, roots, rocks and boulders at streambed and by rivers, 560–1020 m, HA-Cr 143, 316; MS 1197; MS & DPM 3998, 3999, 4068.

*Fissidens kinabaluensis* Z. Iwats.

On soil and termite mount 1100 m, MS & DPM 3886.

*Fissidens nobilis* Griff.

On soil and rocks along trail and river banks, 410–1250 m, DPM 134; HA-Cr 156; MS 1397; MS & DPM 3927, 3966, 4060.

*Fissidens pallidus* Hook. f. & Wilson

On soil and rock, 700–1300 m, DPM 253; HA-Cr 86; MS 989, 1002, 1252; MS & DPM 3952, 4020.

*Fissidens polypodioides* Hedw.

Growing on soil, 1550–1600 m, MS & DPM 2703, 2707.

*Fissidens taxifolius* Hedw.

On stream bank, 680 m, HA-Cr 153.

**Garovagliaceae**

Garovaglia angustifolia Mitt. var. angustifolia

On tree trunks and branches and rotten branches, 770–1770 m, KWL 98; MS 1489; MS & DPM 3975, 4083.

Garovaglia angustifolia var. bogorensis (M. Fleisch.) During

On tree branches, and fallen and rotten logs, 650–1145 m, DPM 255; KWL 11, 67; MS 1205; MS & DPM 3977, 4008.

*Garovaglia brachythecioides* Nog. & Z. Iwats.

On fallen logs and tree branches, 650–1100 m, MS 3896; MS & DPM 4009.

*Garovaglia elegans* (Dozy & Molk.) Hampe ex Bosch & Sande Lac.

On shrub branches, tree trunks and fallen branches, 1320–1700 m, HA-Cr 391; MS 942, 958; MS & DPM 2591, 3850.

Garovaglia plicata (Brid.) Bosch & Sande Lac. subsp. plicata

On climber, shrub and tree trunks, and fallen branches, 1090–1750 m, MS 1389; MS & DPM 2631, 3824, 3852.

**Hookeriaceae**

*Hookeria acutifolia* Hook. & Grev.

On moist to wet boulders beside streams, 1160–1230 m, HA-Cr 383, HA-Cr 398.

**Hypnaceae**

*Ectropothecium eleganti-pinnatum* (Müll. Hal.) A. Jaeger

On a rock, 400 m, DPM 197.

*Ectropothecium ichnotocladum* (Müll. Hal.) A. Jaeger

On shrub leaves, 1440–1600 m, HA-Cr 25; MS & DPM 2676, 2688.

*Ectropothecium moritzii* A. Jaeger

On a decaying log, 1800 m, DPM 60.

*Ectropothecium ptychofolium* N. Nishim.

On tree trunk and base, and shrub branches, 1220–1800 m, DPM 74; HA-Cr 272, 468; MS 983; MS & DPM 2704.

*Ectropothecium striatulum* Dixon ex E.B. Bartram

On a rotten log and stone-wall, 500–1240 m, DPM 176; MS 1396.

Ectropothecium cf. falciforme (Dozy & Molk.) A. Jaeger

On tree trunks, 1800 m, CMK 40, 69; MS & DPM 3802.

It has all the gametophytic characters of the species but sporophytes are not present to confirm its identity.

*Elmeriobryum philippinense* Broth.

On concrete and rocks by road sides in open areas, 1800 m, MS & DPM 3805, 3808, 3809.

*Pseudotaxiphyllum pohliaecarpum* (Sull. & Lesq.) Z. Iwats.

On soil and stone-wall, 600–1400 m, HA-Cr 23, 80; DPM 167, 227.

***Taxiphyllum taxirameum* (Mitt.) M. Fleisch.

On rocks beside stream, 400 m, DPM 188, det. B.C. Tan & B.C. Ho.

Plant small, stems long-creeping, flattened, yellowish-green. Leaves ovate-lanceolate, 1.1–1.2 mm × 0.4 mm, apex gradually finely acuminate, costa short, double and indistinct, inflexed on basal part, margin denticulate. Lamina cells long-rhomboidal, 40–52 μm × 5–7 μm, thin-walled. Sporophyte not seen.

This species has a wide distribution and has been recorded in Java, Malaya, Singapore, Sumatra and the Philippines. It is characterised by its complanate and spreading leaves, with elongated stems as illustrated by Noguchi et al. (1994).

*Trachythecium verrucosum* (A. Jaeger) M. Fleisch.

On soil, 940–1120 m, HA-Cr 246; MS 1226.

*Vesicularia dubyana* (Müll. Hal.) Broth.

On rocks beside stream, 130 m, DPM 284.

*Vesicularia reticulata* (Dozy & Molk.) Broth.

On rocks and stone-wall, 400–450 m, DPM 211, 183, 190.

**Hypnodendraceae**

*Dendro-hypnum beccarii* Hampe

On tree branches, tree trunks and shrub stems, 1370–1800 m, DPM 35, 80, 94; MS 929; MS & DPM 2658, 2583, 3822.

*Dendro-hypnum fuscomucronatum* (Müll. Hal.) N.E. Bell, A.E. Newton & D. Quandt

On boulders and rocks by streams and rivers, 1000–1030 m, G.Gunsalam s.n.; MS 1183, 1184, 1186, 1236.

*Dendro-hypnum milnei* (Mitt.) N.E. Bell, A.E. Newton & D. Quandt

On rocks and boulders by rivers, 680–1230 m, HA-Cr 132, 384; MS 1234, 1227, 1250; MS & DPM 3963, 4092.

Dendro-hypnum subspininervium subsp. arborescens (Mitt.) N.E. Bell, A.E. Newton & D. Quandt

On roots, stumps, tree trunks and boulders, 100–1360 m, DPM 289; HA-Cr 26, 168; MS & DPM 4070.

*Mniodendron dendroides* (Brid.) Wijk & Margad.

On shrub and tree branches, roots and rotten logs, 1240–1800 m, DPM 59, 62, 63, 73, 84; HA-Cr 18, 76, 281; MS & DPM 2685, 3947.

*Touwiodendron diversifolium* (Broth. & Geh.) N.E. Bell, A.E. Newton & D. Quandt

On rotten logs and soils, 1160–1750 m, HA-Cr 380, 402; MS 934, 936, 1404; MS & DPM 2563, 3834, 3945, 4041.

**Hypopterygiaceae**

*Cyathophorum spinosum* (Müll. Hal.) M. Fleisch.

On rotten logs, boulders and shrub trunks, 830–1100 m, MS 1230, 1391; MS & DPM 3988.

*Hypopterygium tamarisci* (Sw.) Brid. ex Müll. Hal.

On rocks, boulders and rotten logs, 500–1600 m, DPM 161; HA-Cr 130, 268; MS 1202; MS & DPM 2686, 3919, 3961.

*Hypopterygium vriesei* Bosch & Sande Lac.

On boulders, 650–830 m, DPM 170; MS & DPM 3986.

*Lopidium struthiopteris* (Brid.) M. Fleisch.

On shrub and tree trunks, roots and rotten logs, 560–1600 m, HA-Cr 244, 299; KWL 86; MS & DPM 2539, 1229, 1394, 2692, 3933.

**Leucobryaceae**

*Bryohumbertia subcomosa* (Dixon) J.-P. Frahm

On rotten logs, stumps, humus and soil, 1350–1850 m, DPM 4; HA-Cr 361; MS & DPM 2647b, 3796, 4048.

*Campylopus comosus* (Schwägr.) Bosch & Sande Lac.

On soil, 600–1150 m, DPM 230; HA-Cr 188.

*Cladopodanthus speciosus* (Dozy & Molk.) M.Fleisch.

On fallen log, 1200 m, MS & DPM 4024.

*Dicranodontium uncinatum* (Harv.) A. Jaeger

On humus and tree trunks, 1800–1900 m, MS & DPM 2642, 2647a, 2653, 3869.

Leucobryum aduncum Dozy & Molk. var. aduncum

On soil, roots, rotten logs, tree trunks and bases, 50–1400 m, DPM 164, 218, 294, 296; KWL 15a; MS & DPM 2545, 2710, 3946, 4050.

Leucobryum aduncum var. scalare (Müll. Hal. ex M. Fleisch.) A. Eddy

On soil, root, rotten logs and tree trunks, 550–1450 m, DPM 175, 154, 220, 231, 232, 243; HA-Cr 135, 372; KWL 81; MS 998, 1430; MS & DPM 2555, 4012.

*Leucobryum arfakianum* Müll. Hal. ex Geh.

On soil, tree trunks and tree bases, 750–1700 m, MS 902, 903, 913, 918, 920, 1245; MS & DPM 2618.

*Leucobryum bowringii* Mitt.

On humus and rotten logs, 1280–1600 m, MS & DPM 2669, 3954, 4045.

*Leucobryum chlorophyllosum* Müll. Hal.

On soil, rotten logs, tree trunks and stump, 400–1150 m, DPM 208, 219, 226, 233, 234, 251; KWL 100a, 53b; MS 1439, 3880; MS & DPM 4015, 4052.

Leucobryum javense (Brid.) Mitt. var. javense

On soil, humus, rotten logs, tree trunks, roots and climbers, 600–1800 m, CMK 46, 70; DPM 27, 31, 224, 228, 236, 246, 250, 258; HA-Cr 400; MS 880, 892, 911; MS & DPM 1253, 2617, 2627, 3867,4091, 4011.

*Leucobryum javense var. cyathifolium (Dixon) T. Yamag.

On humus, 1800 m, MS & DPM 3786.

This is the second report of this variety in Borneo; the first one was from Mount Mulu, Sarawak (Yamaguchi, 1993).

**Leucobryum juniperoideum* (Brid.) Müll. Hal.

On humus and tree bases, 1150–1800 m, MS & DPM 2534, 3870.

In Borneo, this species has been previously reported from Kalimantan and Sarawak. It is widespread in Europe, Macronesia, Madagasca, Turkey, Caucasus, Asia and Malesia (Yamaguchi, 1993).

*Leucobryum sanctum* (Nees ex Schwägr.) Hampe

On humus, soil, root and rotten logs, 50–1400 m, DPM 152, 254, 261, 298; HA-Cr 6; MS 969, 1249, 1263, 1433, 1437, 1440, 1444; MS & DPM 4044, 4047, 4067.

*Leucobryum scabrum* Sande Lac.

On tree trunks, 1400–1800 m, CMK 60, 115; HA-Cr 371.

*Leucobryum sumatranum* Broth. ex M. Fleisch.

On humus, roots and rotten logs, 1100–1620 m, HA-Cr 93; MS 992, 1254; MS & DPM 2586, 3955, 4029.

*Schistomitrium apiculatum* (Dozy & Molk.) Dozy & Molk.

On tree branch, 1150–1770 m, DPM 102; HA-Cr 233.

*Schistomitrium mucronifolium* (A. Braun in Müll. Hal.) M. Fleisch.

On climbers, branches, tree trunks and rotten logs, 1100–1800 m, DPM 21, 32, 34; HA-Cr 79, 183; MS 995; MS & DPM 2578, 2705, 3795, 3835.

*Schistomitrium robustum* Dozy & Molk.

On treelet trunk, 1700 m, MS & DPM 2600, 2604.

**Leskeaceae**

*Claopodium prionophyllum* (Müll. Hal.) Broth.

On boulders and soils, 940–1260 m, HA-Cr 245, 269; MS 1191, 1399; MS & DPM 3960.

*Pseudoleskeopsis zippelii* (Dozy & Molk.) Broth.

On wet boulders in river beds, 1010–1030 m, MS 1193, 1213.

**Leucomiaceae**

*Leucomium strumosum* (Hornsch.) Mitt.

On moist boulder, 1120 m, HA-Cr 258.

**Meteoriaceae**

*Aerobryopsis crispifolia* (Broth. & Geh.) M. Menzel

On tree trunks, and fallen leaves and branches, 850–1425 m, MS 945, 957, 1248.

*Aerobryopsis longissima* (Dozy & Molk.) M. Fleisch.

On twigs, shrub branches, rotten logs and tree trunks, 100–1400 m, DPM 275, 277; HA-Cr 50, 77; MS 949; MS & DPM 3893, 3957.

*Barbella flagellifera* (Cardot) Nog.

On shrub branches, 1600 m, MS & DPM 2667.

***Barbella horridula* Broth.

On a tree trunk, 550 m, DPM 146.

Plants yellowish-green, laxly branched, branches strongly complanate, 1–3 cm long and 5 mm wide, sparsely leaved, often with long flagellae at tips. Branch leaves spreading, narrowly linear-lanceolate, 3.2–3.3 mm × 0.4–0.5 mm, slightly plicate, apex gradually acuminate; margin serulate throughout, recurved on one side at the base; costa single, faint and reaching half of midleaf. Mid lamina cells linear, 108–113 μm × 10–12, rather thin-walled, sometimes with minute 1 (–2) papillae adaxially, long-rhomboidal and thick-walled across insertion. Sporophyte not seen.

This species was previously reported from the Philippines and Sumatra. It can be recognised by its strongly complanate foliation, laxly branched, and linear-lanceolate leaves which are gradually attenuate. The minute papillae are difficult to observe and absent in some leaves. *Barbella horridula* can be distinguished from *Barbella stevensii* (Renauld et Cardot) M. Fleisch. in Broth. from the former hyaline lamina cells (Noguchi, 1976).

*Cryptopapillaria fuscescens* (Hook.) M. Menzel

On shrub and tree branches and treelet trunks, 1070–1700 m, HA-Cr 415; MS 971, 1006, 1233; MS & DPM 3840, 3941.

*Floribundaria floribunda* (Dozy & Molk.) M. Fleisch.

On termite mount and shrub branches, 850–1080 m, HA-Cr 207; MS 928; MS & DPM 3903, 3968.

*Floribundaria intermedia* Thér.

On shrub trunks and leaves, tree branches and buttress, 580–1200 m, MS & DPM 2535, 3987, 4000, 4086.

*Floribundaria pseudofloribunda* M. Fleisch.

On tree trunks, treelet stumps, rocks, boulders, stone-wall, climbers and soil, 550–1090 m, DPM 129, 158, 159, 169; MS 1189, 1231, 1232, 1237; MS & DPM 3915, 3990, 3997.

*Meteoriopsis reclinata* (Müll. Hal.) M. Fleisch.

On a shrub trunk by road side, 900 m, MS & DPM 3913.

*Meteorium polytrichum* Dozy & Molk.

On tree and shrub branches, and fallen logs, 750–1600 m, MS 1192, 1211, 1390; MS & DPM 2665, 2679, 3972, 3984a, 3985, 4094.

*Pseudobarbella ancistrodes* (Renauld & Cardot) Manuel

On a shrub branch by rivers, 850 m, MS & DPM 3984b, 3973.

**Mniaceae**

*Plagiomnium integrum* (Bosch & Sande Lac.) T.J. Kop.

On rocks and tree roots, 640–1240 m, HA-Cr 163, 283.

*Plagiomnium rhynchophorum* (Harv.) T.J. Kop.

On rocks, 850–1800 m, MS 1182; MS & DPM 3861, 3962.

*Plagiomnium succulentum* (Mitt.) T.J. Kop.

On wet boulders beside waterfall, 900–1030 m, HA-Cr 254; MS 1207, 1216; MS & DPM 3922.

**Myuriaceae**

*Oedicladium rufescens* (Reinw. & Hornsch.) Mitt.

On a decaying log, 1500 m, MS 1403.

**Neckeraceae**

*Circulifolium exiguum* (Bosch & Sande Lac.) S. Olsson, Enroth & D. Quandt

On tree trunks, shrub trunks and bases, roots and rotten logs, 500–1021 m, DPM 157; HA-Cr 122; MS 1200, 1201; MS & DPM 4078.

*Circulifolium microdendron* (Mont.) S. Olsson, Enroth & D. Quandt

On stone-walls, boulders, rocks, tree trunks and stumps, 550–1160 m, DPM 143; HA-Cr 129; MS 1244; MS & DPM 3931, 3958.

*Himantocladium cyclophyllum* (Müll. Hal.) M. Fleisch.

On soil, stone-walls, tree trunks, shrub trunks and boulders, 100–1600 m, DPM 171, 178, 263, 312; MS 1442; MS & DPM 2659, 3959.

*Himantocladium plumula* (Nees) M. Fleisch.

On boulders, rocks, and tree trunks, branches, twigs, bases and roots, 100–1145 m, DPM 136, 166, 168, 193, 283, 290; HA-Cr 146, 159, 267; KWL 16, 41, 63; MS 1190; MS & DPM 3910, 3928, 3970, 4085.

*Himantocladium warburgii* (Broth.) M. Fleisch.

On tree trunks, 1300–1650 m, HA-Cr 58, 461.

*Homaliodendron flabellatum* (Sm.) M. Fleisch.

On tree and shrub trunks, and rotten logs, 1100–1700 m, HA-Cr 403; MS 967,1241; MS & DPM 2664, 3846, 4038.

*Neckeropsis gracilenta* (Bosch & Sande Lac.) M. Fleisch.

On climbers, tree trunks, twigs, and decaying logs, 100–1100 m, DPM 280; HA-Cr 169; MS 1228; MS & DPM 3934, 4090.

*Neckeropsis lepineana* (Mont.) M. Fleisch.

On fallen logs and tree trunks, 800–1030 m, MS 1188; MS & DPM 3983, 4089.

*Pinnatella kuehliana* (Bosch & Sande Lac.) M. Fleisch.

On tree roots and stone-walls, 550–1120 m, DPM 150; HA-Cr 249; MS & DPM 3979.

*Pinnatella mucronata* (Bosch & Sande Lac.) M. Fleisch.

On tree trunks and bases, 100–1145 m, DPM 144, 291; HA-Cr 137; KWL 45.

*Thamnobryum incurvum* (Nog.) Nog. & Z. Iwats.

On moist to wet boulders by streams and waterfall, 940–1120 m, HA-Cr 251, 264.

*Thamnobryum subserratum* (Hook. ex Harv.) Nog. & Z. Iwats.

On boulders and wet rocks by waterfall and rivers, 1040–1100 m, MS 1225, 1235, 1238.

*Touwia elliptica* (Bosch & Sande Lac.) S. Olsson, Enroth & D. Quandt

On rocks and boulders, 400–1230 m, DPM 185; HA-Cr 158, 265, 291, 379; MS & DPM 4088.

*Touwia negrosensis* (E.B. Bartram) S. Olsson, Enroth & D. Quandt

On wet boulders, 900 m, MS & DPM 3920.

**Orthotrichaceae**

*Macromitrium cuspidatum* Hampe

On tree trunk, 1150 m, HA-Cr 176.

*Macromitrium longicaule* Müll. Hal.

On boulders, fallen branches, shrub branches and tree trunks, 600–1710 m, MS 944b; MS & DPM 2623, 2711, 3980, 4002, 4007.

*Macromitrium longipilum* A. Braun ex Müll. Hal.

On tree trunks, rotten logs and fallen logs, 1150–1800 m, DPM 106; HA-Cr 227, 228; MS & DPM 3793, 3837, 3800.

*Macromitrium ochraceum* (Dozy & Molk.) Müll. Hal.

On fallen tree branches and tree trunks, 1340–1800 m, MS 944a; MS & DPM 2587, 2691, 3784.

*Macromitrium orthostichum* Nees ex Schwägr.

On boulders and fallen branches, 650–1150 m, HA-Cr 193; MS & DPM 3992, 4093.

*Macromitrium salakanum* Müll. Hal.

On fallen branches, leaves and logs, 400–1380 m, HA-Cr 147; KWL 68, 102; MS 970, 1259, 1260.

*Schlotheimia wallisii* Müll. Hal.

On climber and treelet trunk, 1720–1770 m, MS 1488; MS & DPM 3839.

**Pilotrichaceae**

*Actinodontium rhaphidostegum* (Müll. Hal.) Bosch & Sande Lac.

Growing on rotten logs, shrub branches and fallen branches, 1080–1400 m, HA-Cr 21, 184; MS & DPM 3906.

Callicostella papillata (Mont.) Mitt. var. papillata

On rotten logs, 400–1250 m, DPM 192, 209; HA-Cr 148; MS & DPM 3989, 4051, 4058.

Callicostella papillata var. prabaktiana (Müll. Hal.) Streimann

On rotten logs, boulders and on rocky cliffs, 100–1360 m, DPM 285; HA-Cr 41, 259, 282, 314.

*Cyclodictyon blumeanum* (Müll. Hal.) Kuntze

On moist to wet rocks, 940–1120 m, HA-Cr 243, 294.

*Hookeriopsis utacamundiana* (Mont.) Broth.

On a wet rock by a streamlet, 1240 m, HA-Cr 284.

**Polytrichaceae**

*Dawsonia beccarii* Broth. & Geh.

On rocky cliff in open area, 1780 m, MS & DPM 3877.

*Dawsonia longifolia* (Bruch & Schimp.) Zanten

On soil, 1600–1800 m, DPM 65, 69; MS & DPM 2590, 3940.

Pogonatum cirratum (Sw.) Brid. subsp. cirratum

On soil, 1350–1490 m, MS 1402; HA-Cr 53, 55.

Pogonatum cirratum subsp. fuscatum (Mitt.), Hyvönen

On soil and humus, 750–1340 m, MS 1255, 1256; MS & DPM 4066; MS & DPM 3909; HA-Cr 201; MS 955.

Pogonatum cirratum subsp. macrophyllum (Dozy & Molk.) Hyvönen

On soil, 1220–1800 m, DPM 95; MS & DPM 2561, 3811, 3876, 3944, 4042; MS 991, 954.

*Pogonatum iwatsukii* Touw

On boulders and rocks, 1320–1400 m, MS 930, 940; HA-Cr 350.

*Pogonatum neesii* (Müll. Hal.) Dozy

On soil and boulders in open areas, 680–1800 m, MS & DPM 3871, 3995; MS 951; HA-Cr 54, 120, 424.

*Pogonatum piliferum* (Dozy & Molk.) Touw

On rocks and soil, 400–1360 m, DPM 199; MS & DPM 4018, 4065; HA-Cr 27, 134, 306, 311, 428; MS 990.

*Pogonatum rutteri* (Thér. & Dixon) Dixon

On soil, 1070–1360 m, HA-Cr 28, 394; MS 1003, 1004.

*Pogonatum subtortile* (Müll. Hal.) A. Jaeger

On soil in an open area, 900–1160 m, HA-Cr 204, 406.

**Pottiaceae**

*Chionoloma bombayense* (Müll. Hal.) P. Sollman (1996)

On concrete and stone-wall, 1040–1820 m, DPM 112; MS 1224.

*Barbula javanica* Dozy & Molk.

On thin soil covering wet rocks along stream, 410–1800 m, MS 1461; MS & DPM 3872.

*Barbula consanguinea* (Thwaites & Mitt.) A. Jaeger

On moist stone-wall along river, 1040 m, MS 1223.

*Hyophila involuta* (Hook.) A. Jaeger

Growing on boulders, concrete and stone-walls by waterfall and rivers in open and partially shaded areas, 100–1820 m, DPM 111; DPM 127, 148; DPM 279; HA-Cr 253; MS 1446; MS 1217, 1218; MS & DPM 3923, 4001, 4056.

**Pterobryaceae**

*Calyptothecium recurvulum* (Broth.) Broth.

Hanging on tree trunks by rivers or waterfall, 850–1030 m, MS 1187; MS & DPM 3969, 3974.

*Cryptogonium phyllogonioides* (Sull.) Isov.

On a tree trunk, 940–1120 m, HA-Cr 248.

*Neolindbergia rigida* (Bosch & Sande Lac.) M. Fleisch.

On fallen trees, 1040–1100 m, MS 1195; MS & DPM 3884.

*Neolindbergia rugosa* (Lindb.) M. Fleisch.

On tree trunks and fallen logs, 530–1120 m, HA-Cr 260; MS 1443.

*Pterobryopsis crassicaulis* (Müll. Hal.) M. Fleisch.

On rotten logs and fallen branches, 680–1460 m, HA-Cr 145; MS 1401; MS & DPM 4027.

*Symphysodon neckeroides* Dozy & Molk.

On a tree trunk, 750 m, DPM 256.

*Symphysodontella attenuatula* M. Fleisch.

On tree trunks, 550–1650 m, DPM 145; HA-Cr 438.

*Symphysodontella cylindracea* (Mont.) M. Fleisch.

On shrub trunks, rotten logs, tree trunks and branches, and climbers, 560–1650 m, HA-Cr 300, 439; MS 962, 968, 1405; MS & DPM 2589, 2674, 3894.

*Trachyloma indicum* Mitt.

On rotten logs and tree trunks, 1425–1650 m, HA-Cr 459; MS 960.

**Pylaisiadelphaceae**

*Brotherella falcata* (Dozy & Molk.) M. Fleisch.

On a shrub branch, 1400 m, HA-Cr 339, det. B.C. Tan.

Clastobryum cf. asperrimum (Dixon) B.C. Tan

On a decaying log, 1765 m, DPM 90.

The specimen is similar to the species except for its leaves that are much larger, reaching 1.6 mm long.

*Clastobryum cuculligerum* (Sande Lac.) Tixier

On a fallen branch, 1150 m, HA-Cr 180.

*Clastobryum epiphyllum* (Renauld & Cardot) B.C. Tan & Touw

On rotten twigs and tree trunks, 500–1080 m, DPM 126; MS & DPM 3899a.

**Clastobryum scalare* (Müll. Hal.) Tixier

On tree trunks and branches, leaves and shrub branches, 1800 m, MS & DPM 3790.

In Borneo, this species has been reported from Sarawak and Kalimantan (Dixon 1935; Tixier 1977).

*Isocladiella surcularis* (Dixon) B.C. Tan & Mohamed

On shrub and tree trunks, and roots, 1100–1800 m, CMK 12; HA-Cr 81; KWL 17, 54, 71, 112, 121; MS 993; MS & DPM 2558, 3882, 4019.

*Isopterygium albescens* (Hook.) A. Jaeger

On rotten logs and decaying stump, 1100–1145 m, KWL 8, 12, 70, 85, 105a, 106, 109; MS & DPM 3885.

*Isopterygium bancanum* (Sande Lac.) A. Jaeger

On lianas and fallen trees, 1145 m, KWL 7, 72.

*Isopterygium minutirameum* (Müll. Hal.) A. Jaeger

On a tree base, 500 m, DPM 214.

*Mastopoma armitii* (Broth. & Geh.) Broth.

On shrub and tree trunks and leaves, and rotten logs, 900–1800 m, CMK 44, 47; DPM 39; HA-Cr 11, 203.

*Mastopoma brauniana* (Bosch & Sande Lac.) H. Akiyama

On a tree trunk, 1400 m, HA-Cr 3.

*Mastopoma papillosum* Broth.

Growing on shrub branches, 1730 m, MS & DPM 3838.

*Mastopoma uncinifolium* (Broth.) Broth.

On shrub branches, rotten logs, roots, tree trunks and humus, 1150–1770 m, DPM 82, 101a, 103; HA-Cr 13, 221; MS & DPM 2709, 3833.

*Taxithelium lindbergii* (A. Jaeger) Renauld & Cardot

On tree trunk, branches of shrubs and leaves, 1160–1800 m, HA-Cr 273, 401; MS & DPM 2662, 3866.

*Taxithelium instratum* (Brid.) Broth.

On rotten logs and rocks, 50–800 m, DPM 187, 300, 306; HA-Cr 119, 420.

*Taxithelium isocladum* (Bosch & Sande Lac.) Renauld & Cardot

On a small shrub branch, 1600–1650 m, HA-Cr 455.

*Taxithelium vernieri* (Duby) Besch.

Growing on shrub branches, twigs and fallen tree, 1100–1400 m, KWL 87, 125; HA-Cr 78, 236, 337.

*Trismegistia brachyphylla* M. Fleisch.

On shrub branches and trunks, tree trunks and bases, and rotten logs, 1100–1190 m, HA-Cr 101, 102, 105, 110, 223; MS 999.

Trismegistia calderensis (Sull.) Broth. var. calderensis

On tree branches, shrub trunks and tree stump, 1220–1800 m, MS 937; MS & DPM 1258, 2582, 3797, 3819.

Trismegistia calderensis var. subintegrifolia (Broth.) H. Akiyama

On tree trunks and bases, rotten logs, rotten wood, fallen log, tree branches, soil and rocks, 560–1810 m, HA-Cr 1, 5, 26, 47, 66, 67, 70, 103, 106, 107, 109, 194, 213, 214, 229, 230, 322, 324, 390, 392, 393.

Trismegistia lancifolia var. valetonii (M. Fleisch. ex Dixon) H. Akiyama

On tree trunks and roots, shrub branches, fallen branches, decaying tree, rotten logs and boulders, 610–1150 m, HA-Cr 107, 127, 128, 166, 167, 194, 212, 419, 421; KWL 10, 21, 24, 30, 32, 49, 50, 52b, 57, 61, 65, 74, 82, 88, 92, 96, 99, 103; 113; MS 1438.

Trismegistia panduriformis var. prionodontella (Broth.) H. Akiyama

On shrub branches, tree trunks and bases, rotten logs, decaying wood and boulders, 1100–1770 m, DPM 83, 86, 92, 2672, 4022; HA-Cr 45, 104; MS 935.

**Racopilaceae**

*Racopilum cuspidigerum* (Schwägr.) Ångström

On moist rock beside a stream, 680 m, HA-Cr 131.

*Racopilum laxirete* Broth.

On tree root, 950 m, MS & DPM 3930.

Racopilum spectabile Reinw. & Hornsch. var. spectabile

On shrub leaves and trunks, rotten logs, soils and boulders by rivers, 550–1600 m, DPM 151; HA-Cr 72, HA-Cr 151, 386; MS & DPM 2701; MS 931, 1206, 1398; MS & DPM 3967, 3981.

Racopilum spectabile var. subisophyllum Herzog

On rotten branches and roots, 1600–1800 m, DPM 85; MS & DPM 2678, 3857.

**Regmatodontaceae**

*Regmatodon declinatsus* (Hook.) Brid.

On a boulder beside a stream, 1350 m, MS & DPM 4034.

**Rhizogoniaceae**

*Pyrrhobryum latifolium* (Bosch & Sande Lac.) Mitt.

On tree trunks and bases, and rotten logs, 940–1280 m, HA-Cr 90, 220, 241; MS & DPM 3949.

*Pyrrhobryum spiniforme* (Hedw.) Mitt.

On tree trunks, rotten logs, humus, roots and stone-walls, 400–1780 m, DPM 49, 89, 97, 142, 206, 237, 252; HA-Cr 275, 378; KWL 13, 28, 58, 66, 77, 94, 97, 107; MS 882, 1185; MS & DPM 3832, 3879, 4016, 4017.

*Rhizogonium graeffeanum* (Müll. Hal.) A. Jaeger

On tree trunks and bases, and rotten stumps, 1400–1800 m, HA-Cr 12, 319; MS 901; MS & DPM 3831.

*Rhizogonium lamii* Reimers

On tree buttress and trunks, 1600–1800 m, MS & DPM 2599, 2626, 2668, 3827.

**Sematophyllaceae**

*Acanthorrhynchium papillatum* (Harv.) M. Fleisch.

On tree trunks and tree bases, 560–1145 m, HA-Cr 309; KWL 117; MS & DPM 1436.

*Acroporium adspersum* (Hampe) Broth.

On a tree trunk, 1800 m, CMK 125.

Acroporium convolutum (Sande Lac.) M. Fleisch. var. convolutum

On a tree trunk, 1425 m, MS 963, det. B.C. Tan.

Acroporium convolutum var. elatum (Dixon) B.C. Tan

On rotten logs, 550–1400 m, DPM 153, 239; HA-Cr 296, 310, 368.

*Acroporium diminutum* (Brid.) M. Fleisch.

On tree trunks and branches, decaying logs, climber trunks and shrub branches, 1150–1810 m, CMK 28; DPM 71; HA-Cr 190, 231, 327, 335, 342, 370; MS 877, 893, 959; MS & DPM 2581, 2619, 2629, 2663.

*Acroporium downii* (Dixon) Broth.

On rotten logs and bamboo stumps, 50–1770 m, DPM 101b, 301, 314; KWL 15b; MS & DPM 4063.

*Acroporium johannis-winkleri* Broth.

On tree trunks and branches, roots, rotten logs, fallen logs and shrub branches, 1100–1880 m, CMK 103, 148; DPM 7, 10, 12, 13, 18, 26, 28, 29, 42, 50, 52, 55, 57, 66, 72, 77, 78, 99, 105, 107; HA-Cr 82; MS 987; MS & DPM 2601, 2650, 4021.

*Acroporium lamprophyllum* Mitt.

On leaves, tree trunks, treelet trunks, fallen logs, rotten logs, 1145–1850 m, DPM 9, 24, 38, 44; KWL 18; MS 915; MS & DPM 2648.

**Acroporium macroturgidum* Dixon

On humus, 1800 m, MS & DPM 3818, confirmed by B.C. Tan.

In Borneo, this species has been previously reported from Kalimantan and Sarawak (Suleiman et al. 2006).

Acroporium praelongum var. aciphylloides B.C. Tan

On tree trunks, rotten logs and fallen tree, 1340–1800 m, MS 977; MS & DPM 2607, 3799.

**Acroporium ramicola* (Hampe) Broth.

On tree shrub branches, 1400–1800 m, CMK 6, 90; HA-Cr 367, det. B.C. Tan.

In Borneo, this species has been known only from the type collection from Sarawak (Suleiman et al. 2006).

*Acroporium rigens* (Broth. ex Dixon) Dixon.

On rocks and on rotten logs 600–1370 m, DPM 238; MS 883.

*Acroporium rufum* (Reinw. & Hornsch.) M. Fleisch.

On tree tree trunks and branches, and decaying logs, 1400–1800 m, CMK 76, 133; DPM 8, 33, 51; HA-Cr 73; MS & DPM 2595, 3794.

*Acroporium secundum* (Reinw. & Hornsch.) M. Fleisch.

On shrubs and branches, decaying logs and rotten climbers, 1300–1780 m, DPM 47; HA-Cr 276; MS & DPM 2549, 2609.

Acroporium stramineum (Reinw. & Hornsch.) M. Fleisch. var. stramineum

On tree trunks, decaying fallen log, climbers, shrub branches and fallen branches, 1150–1850 m, DPM 14, 37, 61; CMK 22, 5; HA-Cr 185, 189, 362; MS 912, 953; MS & DPM 2646, 3785.

Acroporium stramineum var. hamulatum (M. Fleisch.) B.C. Tan

On tree trunks, bases and branches, and decaying fallen branches, 1150–1520 m, HA-Cr 177, 186; MS 890, 900, 914; MS & DPM 2553.

Acroporium strepsiphyllum (Mont.) B.C. Tan var. strepsiphyllum

On a fallen log in an open area, 1790 m, DPM 43.

*Chionostomum hainanense* B.C. Tan & Y. Jia

On a tree trunk beside a small pond, 1800 m, MS & DPM 3812, det. K.T. Yong & B.C. Tan.

*Meiothecium hamatum* (Müll. Hal.) Broth.

On tree trunks on shrub branches in open areas, 1150–1800 m, HA-Cr 172, 418; MS & DPM 3778, 3782.

*Meiothecium microcarpum* (Harv.) Mitt.

On boulders in an open area, 600 m, MS & DPM 4004.

**Papillidiopsis malayana* (Dixon) BC. Tan

On a tree branch, 1800 m, MS & DPM 3863, det. B.C. Tan.

In Borneo, this species has been previously reported only from Kalimantan (Dixon, 1935).

*Papillidiopsis ramulina* (Thwaites & Mitt.) W.R. Buck & B.C. Tan

On moist soil in steep galley, 900 m, HA-Cr 205, det. B.C. Tan.

*Papillidiopsis stissophylla* (Hampe & Müll. Hal.) B.C. Tan & Y. Jia

On *Melastoma* branch, shrub branches and trunks, 1600–1800 m, MS & DPM 2690, 3841, 3842, 3865, det. B.C. Tan & K.T. Yong.

Radulina barbonica var. barbonica (Bél.) W.R. Buck

On palm leaves, 385–1400 m, MS 1452; HA-Cr 343.

*Rhaphidostichum piliferum* (Broth.) Broth.

On rotten shrub branch and trunk, 1720 m, MS & DPM 2608, 2612.

***Rhaphidostichum luzonense* (Broth.) Broth.

On boulder s in streambed, 680 m, HA-Cr 149, det. B.C. Tan.

Plants large, yellowish-green, glossy, stems elongate, prostrate, densely branched. Leaves broadly ovate to oblong-ovate, 2.7–3.0 mm × 0.6–0.7 mm, semitubulose, concave, abruptly contracted to a long acuminate apex, ecostate, margin entire below and denticulate at extreme apex. Lamina cells vermicular, 66–98 μm × 5–7 μm, thin-walled, smooth; alar cells large, oval, coloured, inflated. Sporophyte not seen.

This species is characterised by the broadly ovate to oblong-ovate leaves and abruptly contracted into long acuminate apex with denticulate acumen. It was previously only reported from the Philippines (Bartram, 1939; Tan & Iwatsuki, 1991).

*Sematophyllum subpinnatum* (Brid.) E. Britton

On tree and shrub trunks, 900–950 m, MS & DPM 3914, 3935, det. B.C. Tan.

*Trichosteleum boschii* (Dozy & Molk.) A. Jaeger

On shrub trunks and leaves, twigs, tree trunks and branches, rotten logs and rock, 600–1800 m, DPM 87, 100, 242; HA-Cr 35, 39, 142, 407; MS & DPM 2614, 2655, 3829.

*Trichosteleum pseudomammosum* M. Fleisch.

On a tree trunk, 1800 m, CMK 99.

Trichosteleum cf. saproxylophilum (Müll. Hal.) B.C. Tan, W.B. Schofield & H.P. Ramsay

On a tree trunk, 370 m, MS 1261, det. B.C. Tan.

This specimen has all the characteristics of *Trichosteleum saproxylophilum* except for the larger size of the perichaetial leaves (1.7 mm × 0.4 mm) and branch leaves (2.5 mm–2.9 mm × 0.4 mm–0.5 mm).

*Trichosteleum stigmosum* Mitt.

On tree trunks and base, rotten logs and soil, 50–730 m, DPM 215, 240, 248; DPM 297; HA-Cr 318, 307.

Warburgiella cf. breviseta (Broth.) Broth.

On shrub and pandanus leaf, 1400 m, HA-Cr 10, 352, det. B.C. Tan.

The specimens have all the characteristics of the species but the leaf cells are smooth throughout.

*Warburgiella cupressinoides* Müll. Hal. ex Broth.

On a rotten log, 1800 m, MS & DPM 3820.

*Warburgiella circinata* Dixon

On humus, 1400 m, HA-Cr 364, det. B.C. Tan.

**Sphagnaceae**

*Sphagnum cuspidatulum* Müll. Hal.

On humus, 1800 m, MS & DPM 3776, 3788.

*Sphagnum junghuhnianum* Dozy & Molk.

On humus and tree trunk, 1800 m, MS & DPM 3807, 3780, 3804; DPM 19, 54; CMK 78.

*Sphagnum perichaetiale* Hampe

On humus, 1800 m, MS & DPM 3806.

**Symphyodontaceae**

*Chaetomitrium orthorrhynchum* (Dozy & Molk.) Bosch & Sande Lac.

On shrub leaves and branches, tree branches and climber, 310–1600 m, HA-Cr 239; MS 1203, 1262; MS & DPM 2689, 4036.

***Chaetomitrium lancifolium* Mitt.

On a tree branch by a river, partially shaded in secondary lowland forest, 70 m, DPM 307.

Plants medium size for the genus, stems to 3 cm long, irregularly and rather laxly branched. Branches 6–10 mm long, sometimes cuspidate at tips, with clusters of filamentous propagules at tips, sometimes extending to the mid of branches, propagules 1/3 of leaf length. Branch leaves erect to erect-spreading when dry, slightly homomallous, leaves often twisted half above; little altered when wet, except not twisted above; oblong-lanceolate to ovate-lanceolate, concave, 1.3–1.4 mm × 0.4–0.5 mm, apex gradually long-acuminate ending in a narrow point, strongly constricted below apices, costa distinct but short, margin sometimes slightly undulate in upper 1/3, strongly regularly serrate to denticulate to the base, teeth strongly bifid, trifid or multifid. Lamina cells linear, 60 μm × 5 μm in mid-lamina, thick-walled, strongly prorate to spiculose-prorate to the base in adaxial and abaxial sides; alar cells small forming 5–6 short-rectangular cells. Sporophyte not seen.

This species has only been recorded from the Maluku Islands, its type locality. The distinguishing features of this species have been reported recently by Suleiman and Akiyama (2014). It is closely related to *Chaetomitrium papillifolium* Bosch & Sande Lac., differing only in seta length and leaf apex. Based on the type material, this species has a very short seta measuring only 4.4–4.5 cm.

*Chaetomitrium leptopoma* (Schwägr.) Bosch & Sande Lac.

On shrub leaves and branches, 850–1600 m, HA-Cr 234, 325, 334; MS & DPM 2702, 3964.

*Dimorphocladon borneense* Dixon

On ginger leaves by rivers, 748–770 m, MS 1246; MS & DPM 4082, det. B.C. Ho

**Thuidiaceae**

*Pelekium bifarium* (Bosch & Sande Lac.) M. Fleisch.

On a moist stone-wall by a river, 410 m, MS 1456.

*Pelekium velatum* Mitt.

Growing on rocks, tree trunks, rotten logs and stone-walls, 400–900 m, HA-Cr 164; DPM 130; 172, 173, 191; MS & DPM 3918.

*Pelekium versicolor* (Hornsch. ex Müll. Hal.) Touw

On a decaying log, 1050 m, MS 1386.

*Thuidium cymbifolium* (Dozy & Molk.) Dozy & Molk.

On boulders, rocks, stone-wall, rotten logs, stumps, climber and soils, 500–1800 m, DPM 93, 138; HA-Cr 404; MS 1208, 1214, 1220; MS & DPM 2677, 3851, 3860, 3971.

*Thuidium plumulosum* (Dozy & Molk.) Dozy & Molk.

On tree bases, rotten logs, stone-wall, rocks and boulders, 100–680 m, DPM 141, 186, 189, 260; HA-Cr 144, 162; MS 1449, 1451.

Thuidium pristocalyx (Müll.Hal.) A. Jaeger var. pristocalyx

On boulders, tree trunks and buttress, climbers, roots and lianas, 850–1600 m, KWL 29, 37, 38, 39, 40; HA-Cr 88, 344; MS 984; MS & DPM 2670, 3904, 3965.
